# Retraction Note: Circular RNA hsa_circ_0068871 regulates FGFR3 expression and activates STAT3 by targeting miR-181a-5p to promote bladder cancer progression

**DOI:** 10.1186/s13046-026-03866-5

**Published:** 2026-11-05

**Authors:** Weipu Mao, Xin Huang, Longsheng Wang, Ziwei Zhang, Mengnan Liu, Yan Li, Ming Luo, Xudong Yao, Jie Fan, Jiang Geng

**Affiliations:** 1 Department of Urology, Shanghai Tenth People’s Hospital, Tongji University, Shanghai, 200072 China; 2 Department of Urology, Qilu Hospital of Shandong University, Jinan, 250012 China; 3 Department of Pathology, Huashan Hospital, Fudan University, Shanghai, 200040 China

**Retraction Note:** ***J Exp Clin Cancer Res*** **38, 169 (2019)**

https://doi.org/10.1186/s13046-019-1136-9

The Editor-in-Chief has retracted this article. Concerns were raised regarding multiple figures, specifically:

- Figure 3f: certain sections between two panels appear to overlap;
- Figure 5b: STAT3 panel appears to overlap with Actin panel of Fig. 1e of [1].

The source data provided by the authors could not be sufficiently validated. The Editor-in-Chief therefore no longer has confidence in the reliability of the data reported in the article.

None of the authors have responded to any correspondence regarding this retraction.
